# Supplementary material for: Whole-genome landscape of mucosal melanoma reveals diverse drivers and therapeutic targets
Source: Nat Commun. 2019 Jul 18;10:3163. doi: 10.1038/s41467-019-11107-x (PMC6639323; doi:10.1038/s41467-019-11107-x)
Supplement: Supplementary file 1 — Supplementary Information [file 41467_2019_11107_MOESM1_ESM.pdf]

SUPPLEMENTARY INFORMATION

Whole-genome landscape of mucosal melanoma reveals diverse drivers and therapeutic targets

Newell et al.,

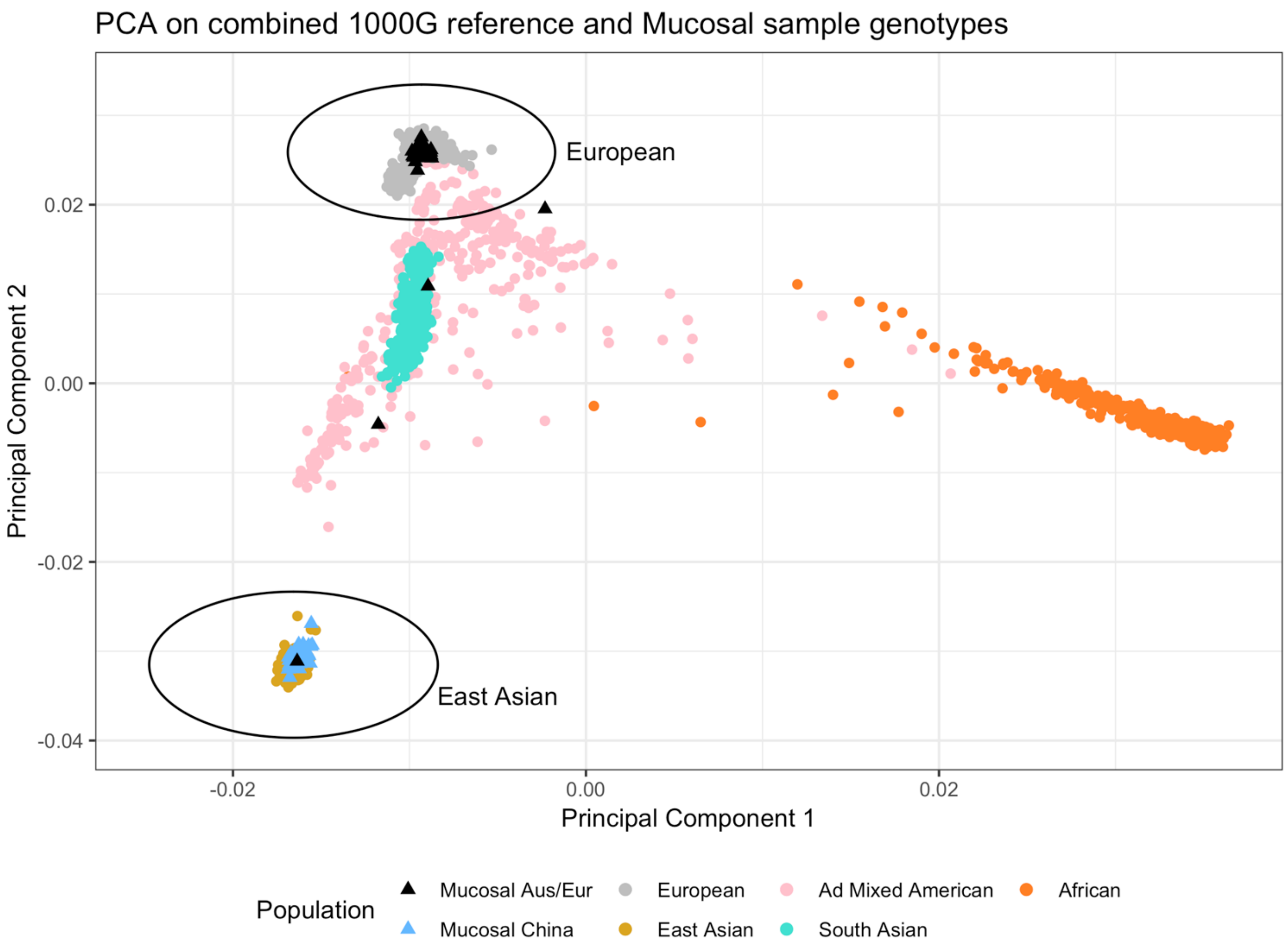

Supplementary Figure 1 | Principal component analysis of mucosal and 1000G sample genotypes Principal component analysis of the genotypes of the 67 whole genome sequenced mucosal samples and 2504 individuals from the 1000 genomes project (phase III) was used to assess ancestry. Each 1000G sample point is coloured by their respective population and x and y axes show principal components 1 and 2. Mucosal samples that were obtained in Australia or European are shown as black triangles and samples obtained in China are shown as light blue triangles.

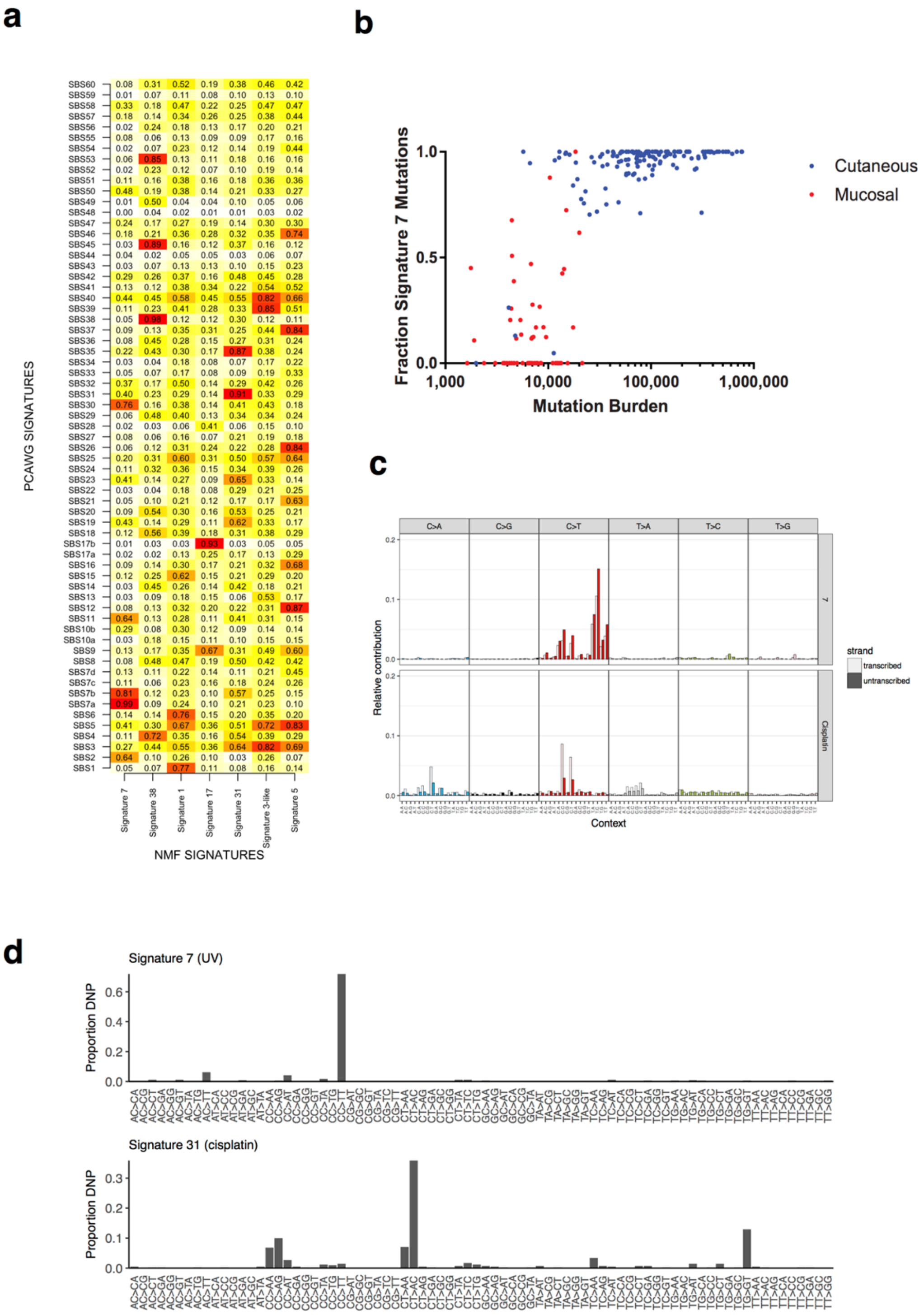

Supplementary Figure 2 | Mutational signature analysis

a) Cosine similarity of NMF signatures with PCAWG signatures. **b)** Comparison of the fraction of signature 7 mutations versus mutation burden in cutaneous and mucosal melanoma subtypes. **c)** Strand bias analysis in signatures 7 (UV) and 31 (cisplatin). **d)** Context of DNPs (dinucleotides) in sample MELA_0765 which has the highest proportion of UV signature in the cohort and sample MELA_0372 which has a high proportion of cisplatin signature 31.

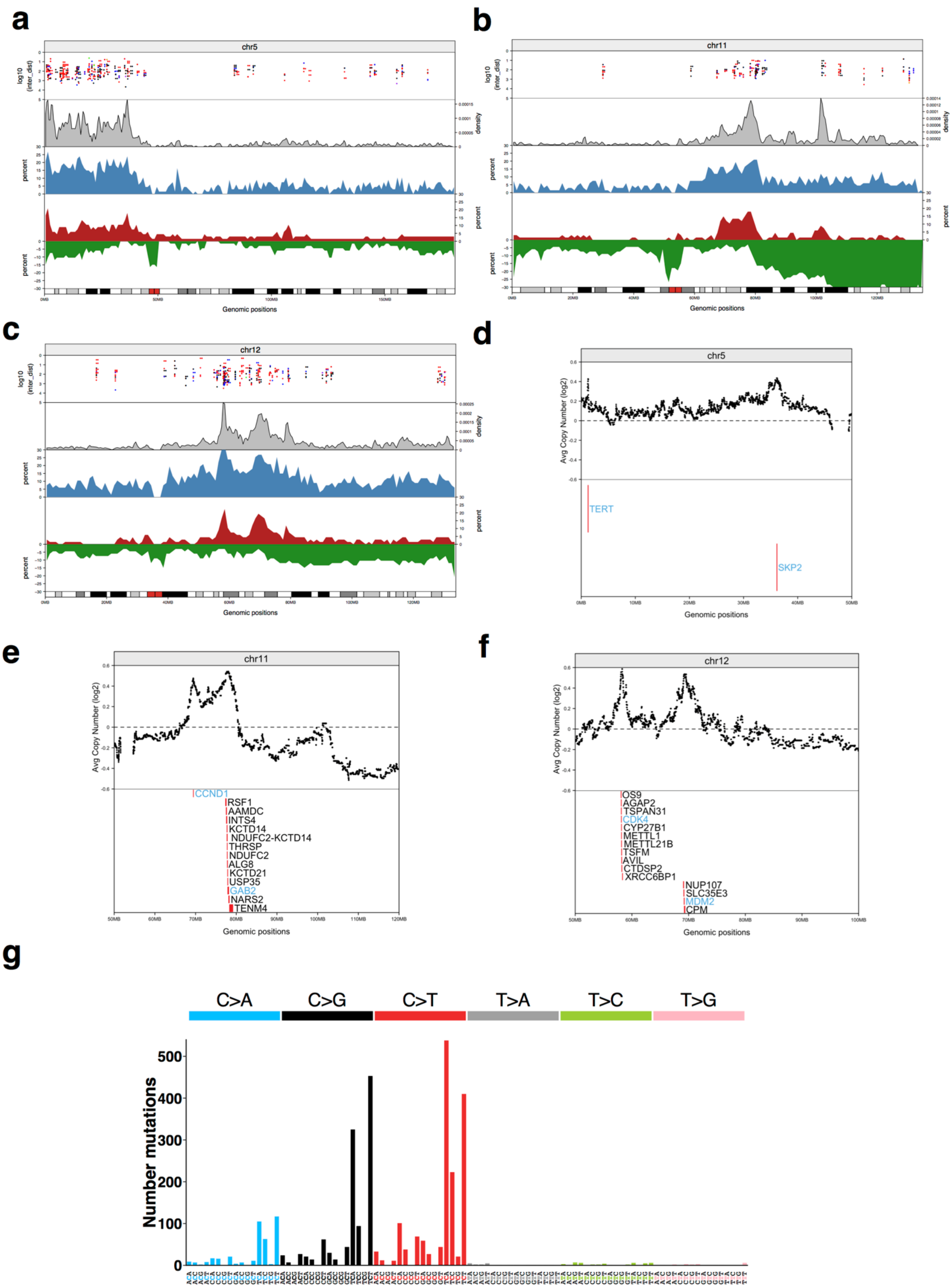
a
chr5
log10 (inter_dist)
density
percent
Genomic positions
b
chr11
c
chr12
d
chr5
Avg Copy Number (log2)
TERT
SKP2
e
chr11
CCND1
RSF1
AAMDC
INTS4
KCTD14
NDUFC2-KCTD14
THRSP
NDUFC2
ALG8
KCTD21
USP35
GAB2
NARS2
TENM4
f
chr12
OS9
AGAP2
TSPAN31
CDK4
CYP27B1
METTL1
METTL21B
TSFM
AVIL
CTDSP2
XRCC6BP1
NUP107
SLC35E3
MDM2
CPM
g
C>A
C>G
C>T
T>A
T>C
T>G
Number mutations

Supplementary Figure 3 | Recurrent regions of complexity

a-c) Chromosomal view of SV events, copy number and kataegis summarised for the cohort (**a**, chr5; **b**, chr11; **c**, chr12). From top to bottom plots display: intermutational distance (log10) of SNVs in kataegic loci (colours for each substitution are: C>A - blue; C>G - black; C>T - red; T>A - grey; T>C - green; T>G - pink); density of SV breakpoints; percent of samples with a SV breakpoint in 1Mb bins; percent of samples with copy number changes in 1Mb bins (amplifications CN≥6 in red, deletions CN0 and 1 in green). **d-f)** Aggregated copy number profiles of amplified regions of chromosomes 5 (**d**), 11 (**e**) and 12 (**f**) in 67 mucosal melanomas. At each position, the mean copy number of the tumors was determined. The y axis shows the log2 copy number change and the axis shows the genomic position. Genes present in highly amplified regions are displayed underneath. Genes highlighted in blue are COSMIC cancer census genes or genes previously associated with amplification and/or chromothripsis in melanomas or other cancers. **g)** Trinucleotide context of SNVs of the 3184 point mutations in 414 kataegis sites which were close to SVs across the cohort resembled the APOBEC signature.

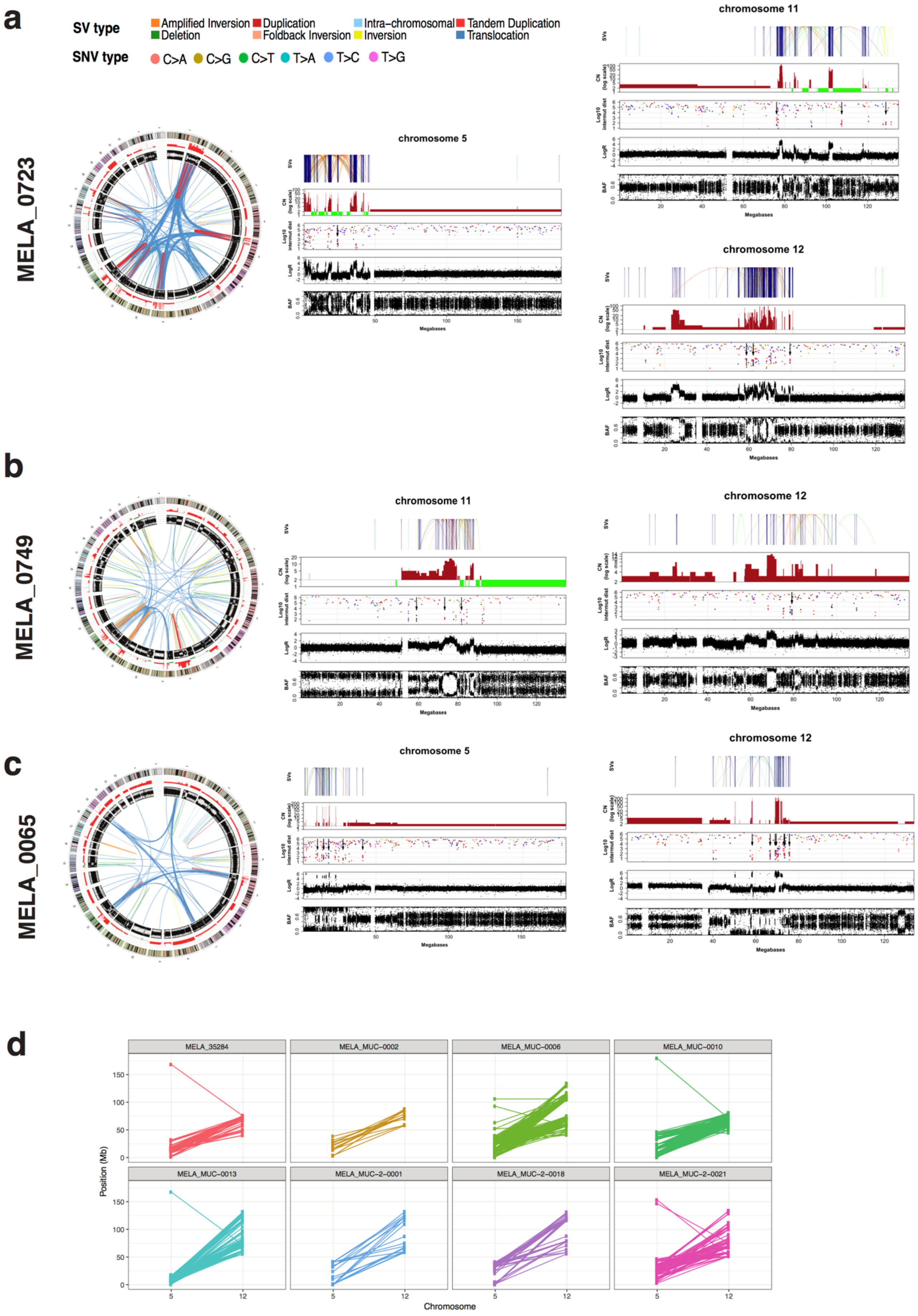
a
SV type
Amplified Inversion
Duplication
Intra−chromosomal
Tandem Duplication
Deletion
Foldback Inversion
Inversion
Translocation
SNV type
C>A
C>G
C>T
T>A
T>C
T>G
MELA_0723
chromosome 5
chromosome 11
chromosome 12
b
MELA_0749
chromosome 11
chromosome 12
c
MELA_0065
chromosome 5
chromosome 12
Megabases
d
MELA_35284
MELA_MUC−0002
MELA_MUC−0006
MELA_MUC−0010
MELA_MUC−0013
MELA_MUC−2−0001
MELA_MUC−2−0018
MELA_MUC−2−0021
Position (Mb)
Chromosome

Supplementary Figure 4 | Examples of complex events in samples.

Circos plots (left) and chromosomal views of complex events (centre, right) in chromosomes 5, 11 and 12 in representative samples MELA_0723 (**a**), MELA_0749 (**b**) and MELA_0065 (**c**). Circos plots show copy number amplifications (red) and deletions (green) in the outer ring, B-allele frequency in the second ring and then structural variants events represented by lines in the inner ring. The color of the lines represent different SV types as indicated in the legend (SV type). Chromosomal views of complex events show SVs in the top plot, copy number amplifications (red) and deletion (green) in the second plot from the top. The third plot from the top shows SNVs in the chromosome, with different colours representing the different types of substitutions as indicated by the legend (SNV type) and the arrows point to regions of kataegis. The fourth plot from top shows LogR ratio and bottom plot shows BAF. **d)** Recurrent translocations between 5p (left side of each plot) and 12q (right side of each plot) in 8 samples. Each line represents a separate translocation event.

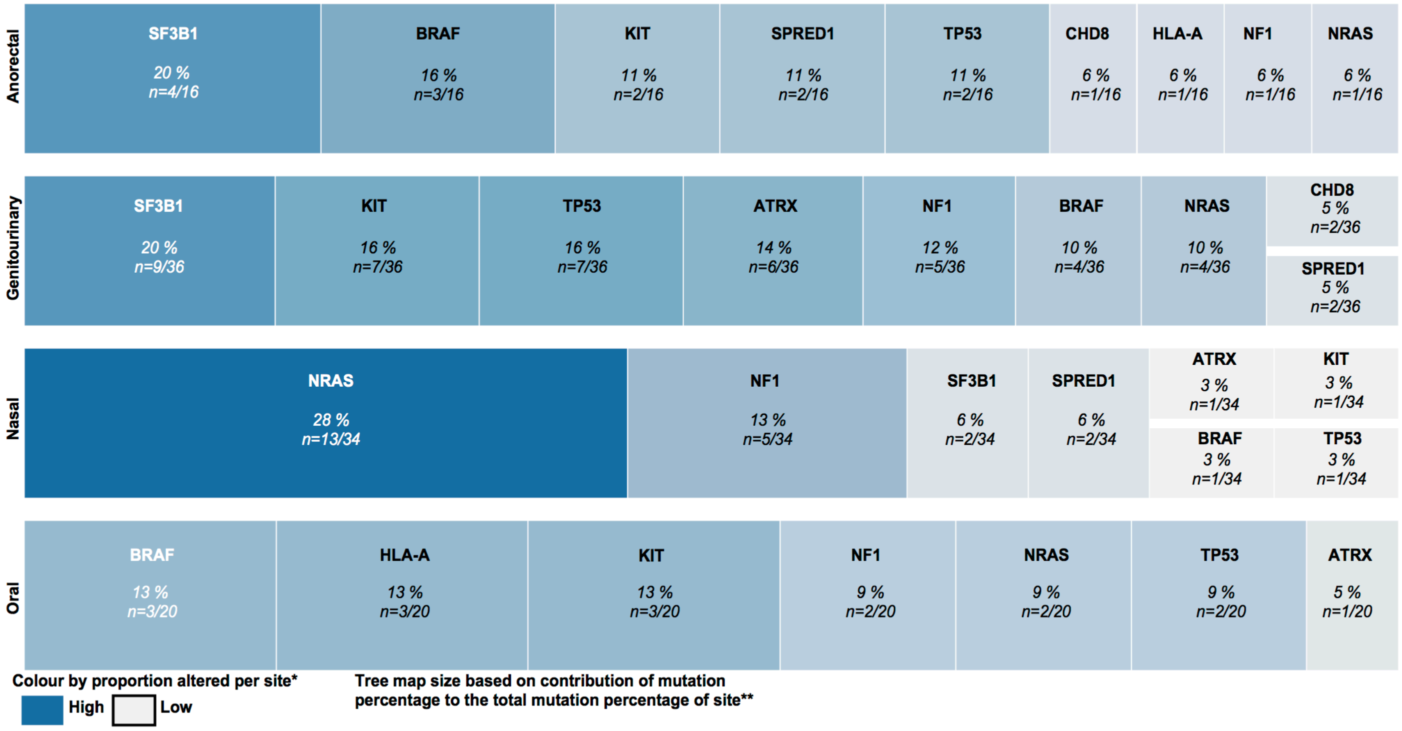

Supplementary Figure 5 | SNV/indel drivers mutation frequency by body site Plot of the percentage and number of SNV/indels drivers by body site, showing the combination of WGS and WES samples. The size of the bar and the colour (darker=higher, lighter=lower) represents the altered fraction. *percentages do not equal 100% as samples contained concurrent mutations and cases lacked a SMG, **tree size expressed as mutation percentage/total mutation percentages per site

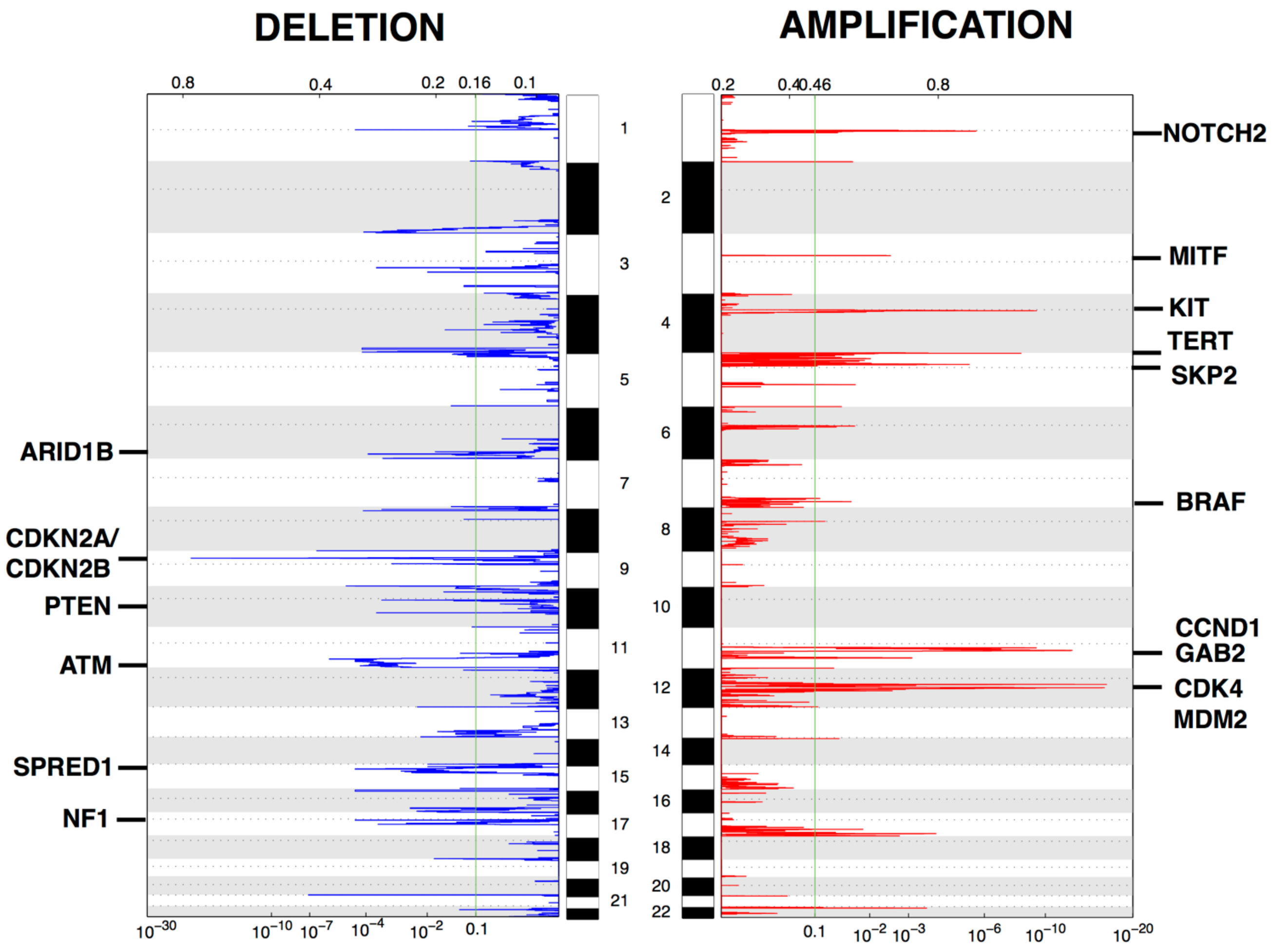

Supplementary Figure 6 | Regions of recurrent copy number gain and loss.

GISTIC analysis was performed to identify recurrent regions of copy number gain and loss (q<0.1). Significant regions of deletion (left, blue) and amplification (right, red) are shown and the highlighted genes in each region are COSMIC cancer census genes, previously reported melanoma genes, genes that were significantly affected by SNV/indels in this cohort or genes in highly amplified regions associated with genomic catastrophes.

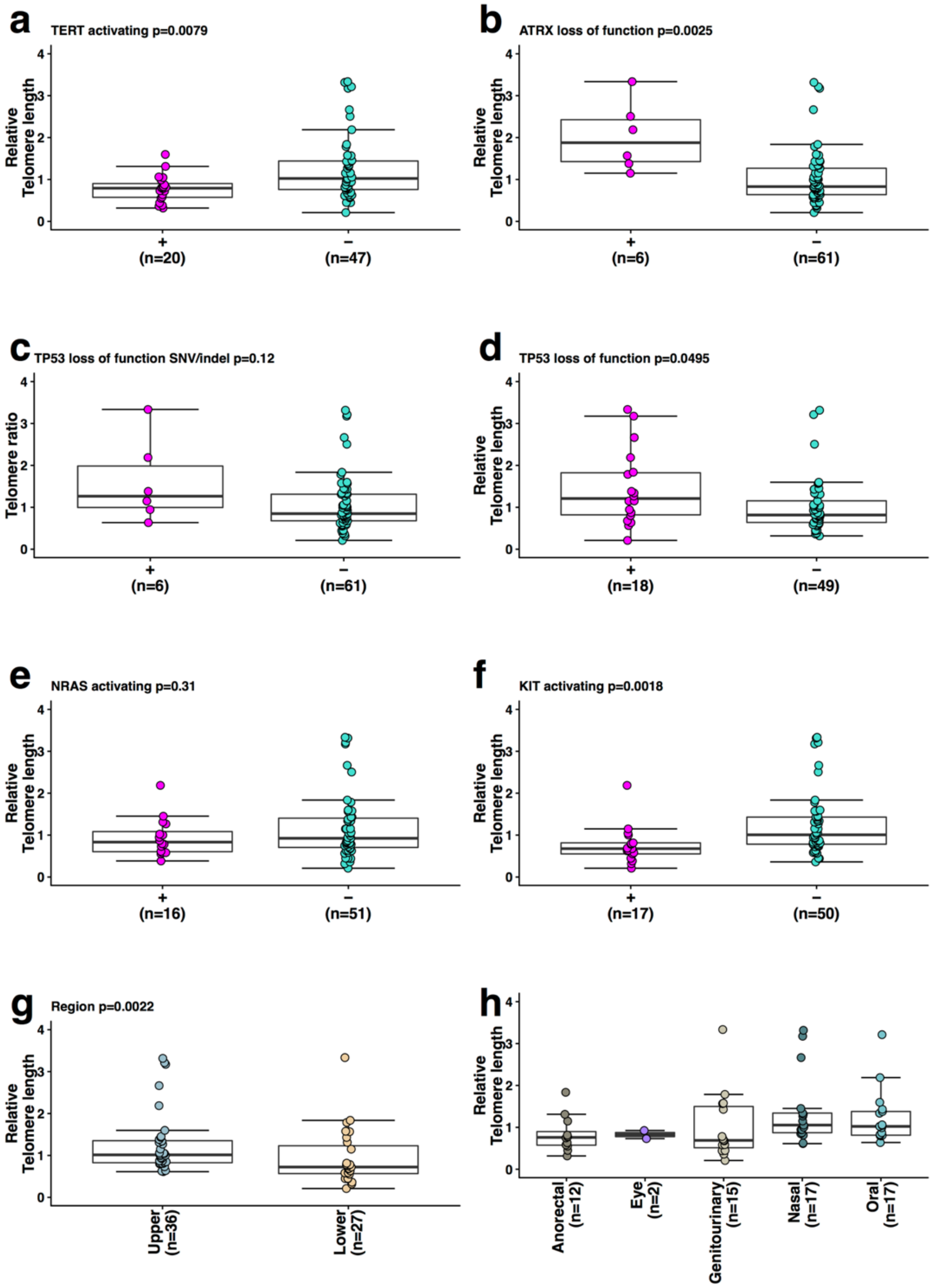

Supplementary Figure 7 | Boxplots of telomere length associations.

Telomere length in: **a)** Samples with or without putatively activating *TERT* mutations (promoter and copy number amplifications), **b)** Samples with or without putative *ATRX* loss of function (SNV/indel and SV) mutations, **c)** Samples with or without putatively *TP53* loss of function SNV/indel mutations, **d)** Samples with or without any type of putative *TP53* loss of function mutation (SNV/indel, copy number 1 and copy neutral LOH), **e)** Samples with or without putatively activating *NRAS* mutations (SNV/indel and copy number amplifications), **f)** Samples with or without putatively activating *KIT* mutations (SNV/indel and copy number amplifications, **g)** Telomere length in upper and lower body sites. **h)** Telomere length in different body regions. In each boxplot, the box boundaries are the 25% to 75% percentile, the median is the centre line and the whiskers represent 1.5 times the inter-quartile range. P-values shown are for two tailed Mann-Whitney tests. Source data are provided as a Source Data file.
