# Supplementary material for: Whole-genome landscape of mucosal melanoma reveals diverse drivers and therapeutic targets
Source: Nat Commun. 2019 Jul 18;10:3163. doi: 10.1038/s41467-019-11107-x (PMC6639323; doi:10.1038/s41467-019-11107-x)
Supplement: Supplementary file 3 — Description of Additional Supplementary Files [file 41467_2019_11107_MOESM3_ESM.pdf]

Description of Additional Supplementary Files

File Name: Supplementary Data 1
Description: Clinico-pathological and sequencing information for 67 mucosal melanoma patients who underwent whole genome sequencing

File Name: Supplementary Data 2
Description: Clinico-pathological and sequencing information for 45 mucosal melanoma patients who underwent whole exome sequencing from FFPE

File Name: Supplementary Data 3
Description: SNV/indel coding mutations

File Name: Supplementary Data 4
Description: Significantly mutated genes

File Name: Supplementary Data 5
Description: Predicted in frame gene fusions

File Name: Supplementary Data 6
Description: Clinically actionable mutations
