# Supplementary material for: Whole-genome landscape of mucosal melanoma reveals diverse drivers and therapeutic targets
Source: Nat Commun. 2019 Jul 18;10:3163. doi: 10.1038/s41467-019-11107-x (PMC6639323; doi:10.1038/s41467-019-11107-x)
Supplement: Supplementary file 10 — Reporting Summary [file 41467_2019_11107_MOESM10_ESM.pdf]

nature research

Corresponding author(s): Richard Scolyer

Last updated by author(s): Mar 26, 2019

Reporting Summary

Nature Research wishes to improve the reproducibility of the work that we publish. This form provides structure for consistency and transparency in reporting. For further information on Nature Research policies, see Authors & Referees and the Editorial Policy Checklist.

Statistics

For all statistical analyses, confirm that the following items are present in the figure legend, table legend, main text, or Methods section.

n/a	Confirmed	
☐	☒	The exact sample size (*n*) for each experimental group/condition, given as a discrete number and unit of measurement
☐	☒	A statement on whether measurements were taken from distinct samples or whether the same sample was measured repeatedly
☐	☒	The statistical test(s) used AND whether they are one- or two-sided<br>*Only common tests should be described solely by name; describe more complex techniques in the Methods section.*
☒	☐	A description of all covariates tested
☒	☐	A description of any assumptions or corrections, such as tests of normality and adjustment for multiple comparisons
☐	☒	A full description of the statistical parameters including central tendency (e.g. means) or other basic estimates (e.g. regression coefficient) AND variation (e.g. standard deviation) or associated estimates of uncertainty (e.g. confidence intervals)
☒	☐	For null hypothesis testing, the test statistic (e.g. *F*, *t*, *r*) with confidence intervals, effect sizes, degrees of freedom and *P* value noted<br>*Give P values as exact values whenever suitable.*
☒	☐	For Bayesian analysis, information on the choice of priors and Markov chain Monte Carlo settings
☒	☐	For hierarchical and complex designs, identification of the appropriate level for tests and full reporting of outcomes
☒	☐	Estimates of effect sizes (e.g. Cohen's *d*, Pearson's *r*), indicating how they were calculated

Our web collection on statistics for biologists contains articles on many of the points above.

Software and code

Policy information about availability of computer code

Data collection	No code was used for data collection
Data analysis	The following tools were used: qSNP (version 2.0), GATK HaplotypeCaller (version 3.3-0), SnpEff (version 4.0e build 2014-09-13),Cutadapt (version 1.9), BWA-MEM (version 0.7.12),SAMtools (version 1.1),Picard MarkDuplicates (version 1.129), Biobambam (version 2.0.18), qProfiler (version 1) qCoverage (version 0.7pre), MuTect (v1.1.7), MAC (v1.2), Strelka (v1.0.15), plink (version 1.90b6.8), plinkQC R package (0.2.0), R (v3.4.4), MutationalPatterns R packages (v1.4.3), SignatureEstimation R package (1.0.0), lollipops (v1.3.2), ConsensusClusterPlus R package (v1.42.0),OncodriveFML (v2.0), Music2 (v0.2), dndScv R package (v0.0.0.9), 20/20+ (v1.1.3), Intogen Pipeline for OncodriveFM and OncodriveClust (v3.0.8), ascatNgs (v4.0.1), qSV (v0.3), GISTIC (v2.0.22), qMotif (v1.2), Matlab (version R2016a), MutSigCV (v1.4)

For manuscripts utilizing custom algorithms or software that are central to the research but not yet described in published literature, software must be made available to editors/reviewers. We strongly encourage code deposition in a community repository (e.g. GitHub). See the Nature Research guidelines for submitting code & software for further information.

Data

Policy information about availability of data

All manuscripts must include a data availability statement. This statement should provide the following information, where applicable:

- Accession codes, unique identifiers, or web links for publicly available datasets
- A list of figures that have associated raw data
- A description of any restrictions on data availability

The raw sequencing data for whole genome sequenced samples are available in the European Genome-phenome Archive (EGA) under study accession EGAS00001001552. The raw sequencing data for the UK/USA WES samples are available for download from the EGA under study accession EGAS00001001115. The source data underlying Fig 1c and Supplementary Fig 5 are provided as a Source Data file.

Field-specific reporting

Please select the one below that is the best fit for your research. If you are not sure, read the appropriate sections before making your selection.

☒ Life sciences ☐ Behavioural & social sciences ☐ Ecological, evolutionary & environmental sciences

For a reference copy of the document with all sections, see nature.com/documents/nr-reporting-summary-flat.pdf

Life sciences study design

All studies must disclose on these points even when the disclosure is negative.

Sample size	We sequenced 67 samples using whole genome analysis and had 45 FFPE samples for validation of SNVs/indels found. As mucosal melanoma is a rare subtype of melanoma, sample size is limited by the availability of appropriate samples. Nonetheless this is the largest study to date of whole genome sequenced melanoma samples
Data exclusions	No data were excluded from the analyses unless a specific parameter eg sample site was being compared. Samples with unknown data for that variable were excluded for that analysis.
Replication	45 FFPE samples were used to validated the SNV/indel mutations findings from the analysis of 67 whole genome samples.
Randomization	Samples were not randomized
Blinding	There was no sample blinding

Reporting for specific materials, systems and methods

We require information from authors about some types of materials, experimental systems and methods used in many studies. Here, indicate whether each material, system or method listed is relevant to your study. If you are not sure if a list item applies to your research, read the appropriate section before selecting a response.

Materials & experimental systems

n/a	Involved in the study	
☒	☐	Antibodies
☒	☐	Eukaryotic cell lines
☒	☐	Palaeontology
☒	☐	Animals and other organisms
☐	☒	Human research participants
☒	☐	Clinical data

Methods

n/a	Involved in the study	
☒	☐	ChIP-seq
☒	☐	Flow cytometry
☒	☐	MRI-based neuroimaging

Human research participants

Policy information about studies involving human research participants

Population characteristics	See Supplementary Data 1 and 2 for all donor characteristics
Recruitment	WGS Samples were obtained from biospecimen bank of Melanoma Institute Australia (MIA) (n=24), Peking University Cancer Hospital & Institute, Beijing, China (n=39), the Department of Surgery, Skåne University Hospital, Sweden (n=3), and the Biobank of the University Research Priority Program in translational cancer research (URPP) at the University of Zurich Hospital, Switzerland (n=1). FFPE tissue were from three clinical centers: University of Michigan, University of Edinburgh and University of California, San Francisco. All tissues and bloods form part of prospective collections of samples accrued with written informed patient consent and institutional review board approval at the above institutions.
Ethics oversight	All tissues and bloods form part of prospective collections of samples accrued with written informed patient consent and institutional review board approval at the above institutions.

Note that full information on the approval of the study protocol must also be provided in the manuscript.
